# Sequence-specific inhibition of reverse transcription by recombinant CRISPR/dCas13a ribonucleoprotein complexes *in vitro*

**DOI:** 10.1093/biomethods/bpab009

**Published:** 2021-04-19

**Authors:** Toshitsugu Fujita, Shoko Nagata, Miyuki Yuno, Hodaka Fujii

**Affiliations:** 1 Department of Biochemistry and Genome Biology, Hirosaki University Graduate School of Medicine, 5 Zaifu-cho, Hirosaki, Aomori 036-8562, Japan; 2 Chromatin Biochemistry Research Group, Combined Program on Microbiology and Immunology, Research Institute for Microbial Diseases, Osaka University, 3-1 Yamadaoka, Suita, Osaka 565-0871, Japan; 3 Research Division, Kobe Research Center, Epigeneron, Inc., CoLaborator Kobe 602, Kobe International Business Center, 5-5-2 Minatojima Minamimachi, Chuo-ku, Kobe 650-0047, Japan

**Keywords:** CRISPR, Cas13a, dCas13a, reverse transcription, RNA editing

## Abstract

The clustered regularly interspaced short palindromic repeats (CRISPR) system is widely used for genome editing because of its ability to cleave specific DNA sequences. Recently, RNA-specific CRISPR systems have been reported. CRISPR systems, consisting of a guide RNA (gRNA) and a nuclease-dead form of Cas13a (dCas13a), can be used for RNA editing and visualization of target RNA. In this study, we examined whether a recombinant CRISPR/dCas13a ribonucleoprotein (RNP) complex could be used to inhibit reverse transcription (RT) in a sequence-specific manner *in vitro*. Recombinant *Leptotrichia wadei* dCas13a was expressed using the silkworm-baculovirus expression system and affinity-purified. We found that the CRISPR/dCas13a RNP complex, combined with a chemically synthesized gRNA sequence, could specifically inhibit RT of *EGFR* and *NEAT1*, but not nonspecific RNA. Thus, the CRISPR/dCas13a RNP complex can inhibit RT reactions in a sequence-specific manner. RT inhibition by the CRISPR/dCas13a system may be useful to assess target binding activity, to discriminate RNA species retaining target sequences of gRNA, or to suppress RT from undesirable RNA species.

## Introduction

The clustered regularly interspaced short palindromic repeats (CRISPR) and associated Cas protein system are part of the adaptive immune machinery of bacteria and archaea to defend against foreign DNA elements [1, 2]. In particular, the CRISPR/Cas9 system from *Streptococcus pyogenes* is widely used for genome editing [3, 4]. In the CRISPR/Cas9 system, a nucleotide sequence complementary to a guide RNA (gRNA) sequence is cleaved, which can occasionally introduce nucleotide mutations in a target cell.

Recently, the CRISPR/Cas13a system was identified to target RNA and has since been used for RNA editing [5, 6]. Because CRISPR/Cas13a can distinguish a single-nucleotide difference in a target sequence, it can also be applied to detect and discriminate subtypes of RNA viruses or single-nucleotide mutations in transcribed genes of cancer cells [7, 8]. In addition to RNA editing and detection, the nuclease-dead form of Cas13a (dCas13a) can be used to tag and locate RNA in cells [9]. Thus, CRISPR/Cas13a is widely applicable to RNA biology.

**Figure 1:**
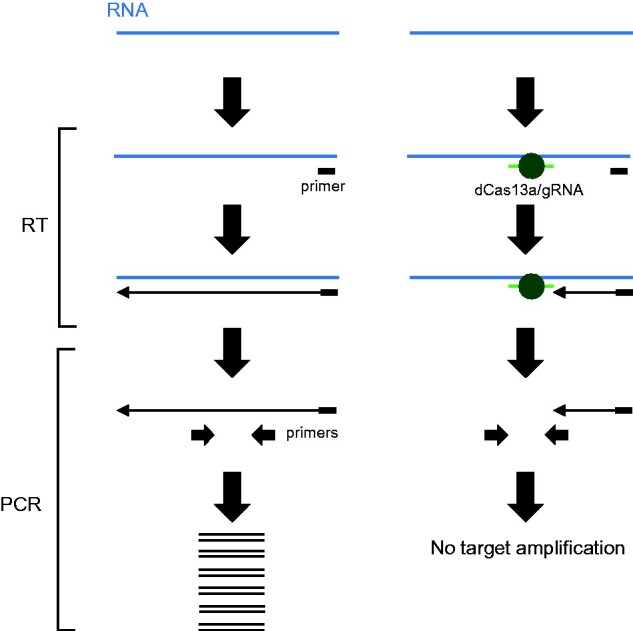
application of the recombinant dCas13a/gRNA RNP complex for sequence-specific inhibition of RT. In this schema, the recombinant CRISPR/dCas13a RNP complex functions as an RT blocker in a sequence-specific manner. The blocking effect can be evaluated by subsequent PCR analysis

In this study, it was examined whether CRISPR/dCas13a can be used as a sequence-specific blocker, by inhibiting reverse transcription (RT) with reverse transcriptases in a test tube (Fig. 1). To achieve this, a recombinant *Leptotrichia wadei* dCas13a (Lw-dCas13a) was produced. It was determined that a ribonucleoprotein (RNP) complex, consisting of the recombinant dCas13a protein and synthetic gRNA, can be used as a sequence-specific blocker for RT reactions. This RT inhibition system may be used to estimate whether designed gRNAs can be functional *in vitro* or suppress RT of undesirable RNA species.

## Materials and methods

### Cell lines

The human lung cell lines, MRC-5 and NCI-H1299, were purchased from American Type Culture Collection (Manassas, VA, USA). MRC-5 was cultured in Eagle's minimal essential medium (E-MEM) (Wako, Osaka, Japan) supplemented with 10% fetal bovine serum (FBS). NCI-H1299 was cultured in RPMI-1640 (Wako, Osaka, Japan) supplemented with 10% FBS.

### Preparation of a recombinant dCas13a protein

To produce a recombinant dCas13a (r3xFLAG-dCas13a-D) protein, an expression construct of Lw-dCas13a [9] was first generated. Next, the recombinant Lw-dCas13a protein was expressed using the silkworm–baculovirus expression system, then affinity-purified (ProCube^®^, http://procube.sysmex.co.jp/eng/) (Sysmex Corporation, Hyogo, Japan) as described previously [10]. The final protein concentration was 1.2 µg/µl.

### Preparation of dCas13a/gRNA RNP complexes

gRNAs were synthesized by Gene Design, Inc (Osaka, Japan) (Supplementary Table S1). The synthesized gRNAs were diluted with nuclease-free water to a final concentration of 10 µM. 1 µl of gRNA was mixed with 3 µl of nuclease-free water and then incubated at 100°C for 2 min and cooled to room temperature. To make the dCas13a/gRNA RNP complex, 0.4 µl of recombinant dCas13a protein (0.48 µg), 0.92 µl of gRNA, and 3.68 µl of nuclease-free water were mixed in a total volume of 5 µl.

### RT reactions

Total RNA was extracted from MRC-5 or NCI-H1299 using Isogen II (Nippon Gene, Tokyo, Japan). Total RNA from either of these cell lines was used throughout this study. RT was performed using ReverTra Ace qPCR RT Kit (Toyobo, Shiga, Japan). Briefly, 1 µl of the total RNA (100 ng for Supplementary Figs S4 and S5 or 10 ng for others), 2 µl of 5× RT buffer, 0.5 µl of enzyme mix, 0.5 µl of oligo(dT)20 primer (FSK-201, Toyobo), 5 µl of the dCas13a/gRNA RNP complex, and 1 µl of nuclease-free water were mixed in a total volume of 10 µl. Reaction mixtures were incubated for 15 min at 37°C, followed by 5 min at 98°C. After the RT reaction, 20 µl of nuclease-free water was added (total 30 µl).

### Real-time PCR analysis

Real-time PCR was performed using THUNDERBIRD^®^ SYBR qPCR Mix (Toyobo). Briefly, 2 µl of cDNA, 0.5 µM of each primer, and nuclease-free water were combined with THUNDERBIRD^®^ SYBR qPCR Mix in a total volume of 10 µl following the manufacture’s protocol. The PCR cycling conditions using CFX Connect^TM^ (Bio-Rad, Hercules, CA, USA) were as follows: 1 cycle of 95°C for 1 min; 40 cycles of 95°C for 15 s; and 60°C for 30 s. Primers used in this study are shown in Supplementary Table S1.

### Statistical analysis

Statistical analysis was performed with the Prism software 6 (GraphPad, San Diego, CA, USA) using one-way analysis of variance.

## Results and discussion

**Figure 2:**
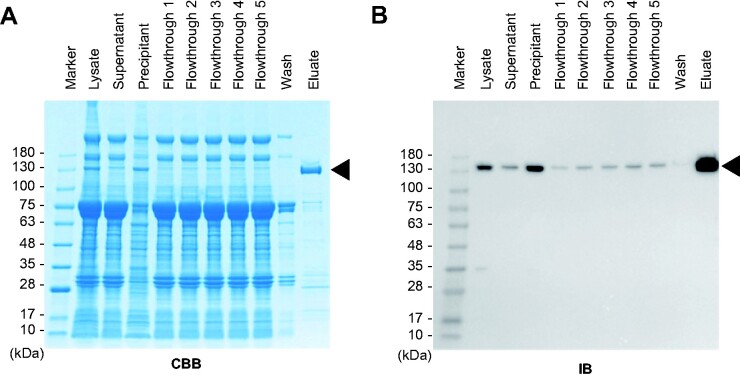
preparation of a recombinant dCas13a protein. (A and B) Preparation of r3xFLAG-Lw-dCas13a-D, a recombinant dCas13a protein. Proteins were subjected to SDS-PAGE followed by Coomassie Brilliant Blue staining (CBB) (A) or immunoblot analysis with anti-Dock Ab (IB) (B). Marker: molecular weight marker. The lysate, supernatant, and precipitant were all products of silkworm pupal homogenates. The flowthrough, wash, and eluate samples were products of affinity purification. The position of r3xFLAG-Lw-dCas13a-D is shown with arrowheads

First, a recombinant *L. wadei* dCas13a (r3xFLAG-Lw-dCas13a-D) protein was generated using the silkworm–baculovirus expression system. r3xFLAG-Lw-dCas13a-D could be purified without visible degradation (Fig. 2A and B).

Next, it was examined whether the RT reactions were disturbed in the presence of the purified r3xFLAG-Lw-dCas13a-D alone. Using 10 ng of the MRC-5 total RNA, RT reactions were performed in the presence or absence of the protein. The synthesized cDNA products from the RT reactions were then used for real-time PCR. It was determined that 0.24 µg of r3xFLAG-Lw-dCas13a-D did not inhibit 10 µl of RT reactions of *EGFR*, a target RNA in this study (see below) (Supplementary Fig. S1). However, 0.96 µg of the protein showed modest nonspecific inhibition of RT reactions (Supplementary Fig. S1). Therefore, hereafter, 0.48 µg of the dCas13a protein was used for RT reactions (see Materials and Methods section).

**Figure 3:**
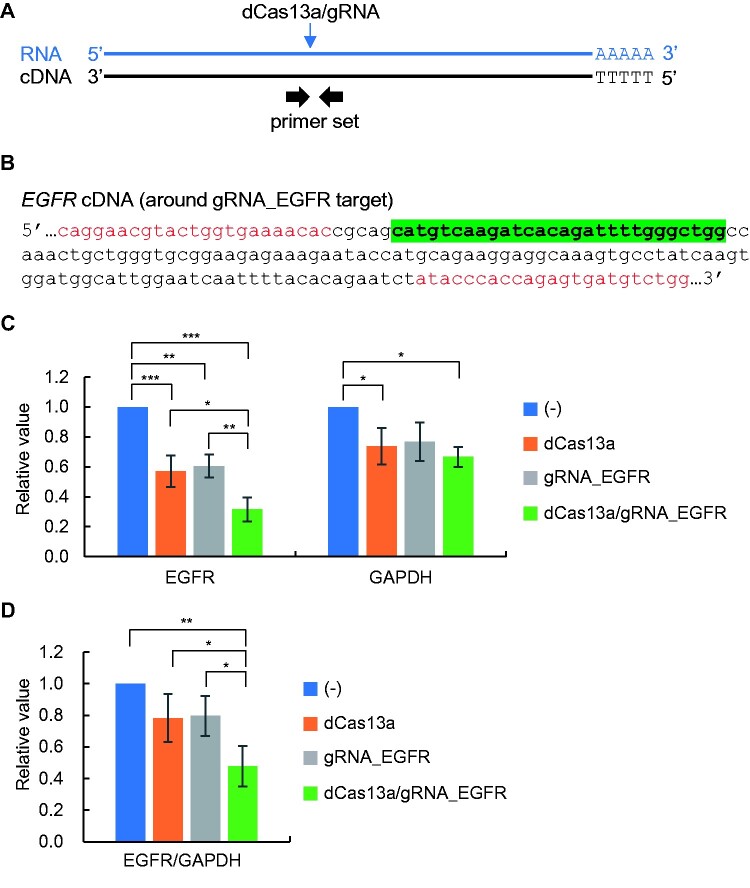
sequence-specific inhibition of RT by the recombinant dCas13a/gRNA_EGFR RNP complex. (A) Schematic diagram of target and primer positions. (B) Primer positions are shown in red, and the cDNA sequence corresponding to gRNA targeting *EGFR* RNA is highlighted. (C) RT reactions in the presence of the dCas13a/gRNA_EGFR RNP complex, the dCas13a protein alone, or the gRNA_EGFR alone. To evaluate RT blocking effects, the cDNA was subjected to real-time PCR to amplify *EGFR* and *GAPDH*. (D) Results with normalization to *GAPDH*. Error bars represent the standard deviation of three independent RT experiments (C and D). *P*-values (* < 0.05, ** < 0.01, *** < 0.001) are shown when statistical significance was achieved

Next, it was examined whether the dCas13a/gRNA RNP complex could inhibit RT reactions in a sequence-specific manner. To this end, a reported gRNA targeting the wild-type *EGFR* sequence (gRNA_EGFR) [7] was used (Fig. 3A and B;Supplementary Table S1). Because we previously confirmed that wild-type *EGFR* mRNA is expressed in various lung cells, such as a normal lung fibroblast cell line, MRC-5, and a nonsmall cell lung carcinoma cell line, NCI-H1299 [11], total RNA was extracted from MRC-5 and used as a model RNA. RT reactions were performed using total RNA and the oligo(dT) primer in the presence of the dCas13a/gRNA_EGFR RNP complex, the dCas13a protein alone, or the gRNA alone. cDNA was subjected to real-time PCR to evaluate their RT-blocking effects. In this study, a pair of primers between which the target site is located was used for real-time PCR because RT proceeds from somewhere in RNA even in the absence of primers (primer-independent cDNA synthesis) [12, 13] (Supplementary Fig. S2A) and a primer set upstream of the target site may obscure the evaluation of RT-blocking effect (Supplementary Fig. S2). As shown in Fig. 3C, decreased PCR amplification of *EGFR* occurred when the cDNA was synthesized with the dCas13a/gRNA_EGFR RNP complex. Although the dCas13a protein or the gRNA alone showed decreased *EGFR* PCR amplification*,* probably in a nonspecific manner, the dCas13a/gRNA RNP complex was most effective to suppress PCR amplification. The dCas13a/gRNA_EGFR RNP complex did not specifically suppress PCR amplification of *GAPDH* included as a negative control target, compared with the dCas13a protein or the gRNA alone (Fig. 3C). Normalization of *EGFR* to *GAPDH* demonstrated a significant decrease in PCR amplification by the dCas13a/gRNA_EGFR RNP complex (Fig. 3D). These results suggest that the dCas13a/gRNA_EGFR RNP complex is capable of target-specific inhibition of RT reactions.

**Figure 4:**
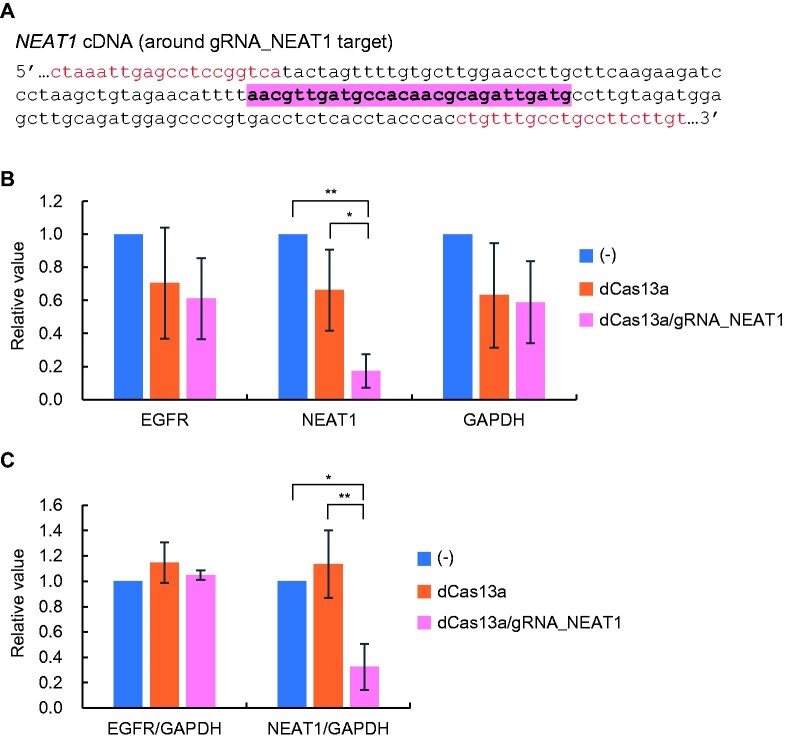
sequence-specific inhibition of RT by the recombinant dCas13a/gRNA_NEAT1 RNP complex. (A) Target and primer positions. Primer positions are shown in red, and the cDNA sequence corresponding to gRNA targeting *NEAT1* RNA is highlighted. (B) RT reactions in the presence of dCas13a/gRNA_NEAT1 RNP complex. To evaluate the RT-blocking effect of the dCas13a/gRNA_NEAT1 RNP complex, the cDNA was subjected to real-time PCR to amplify *NEAT1*, *EGFR*, and *GAPDH*. (C) Results with normalization to *GAPDH*. Error bars represent the standard deviation of three independent RT experiments (B and C). *P*-values (* < 0.05, ** < 0.01) are shown when statistical significance was achieved

To validate sequence-specific inhibition of RT by the dCas13a/gRNA RNP complex, another gRNA was designed to target a noncoding RNA. In this study, we targeted *NEAT1* [14] as a model RNA (Fig. 4A and Supplementary Table S1). When the cDNA was synthesized with the dCas13a/gRNA_NEAT1 RNP complex and then subjected to real-time PCR with a pair of primers between which the target site is located, we found decreased amplification of *NEAT1*, but no significant differences with *EGFR* nor *GAPDH* (Fig. 4B). When normalized to *GAPDH*, a decrease in *NEAT1* PCR amplification but not *EGFR* was demonstrated by the dCas13a/gRNA_NEAT1 RNP complex (Fig. 4C). We also observed sequence-specific inhibition of RT reactions by the dCas13a/gRNA_EGFR or gRNA_NEAT1 RNP complex when total RNA extracted from NCI-H1299 was tested as a template (Supplementary Figs S3 and S4). We also tested another gRNA targeting a *NEAT1* sequence (gRNA_NEAT1_2). As shown in Supplementary Fig. S5, dCas13a/gRNA_NEAT1_2 RNP demonstrated strong inhibition of RT reactions for *NEAT1* in a sequence-specific manner although moderate nonspecific inhibition effect to *GAPDH* was also observed. Together, these results showed that the dCas13a/gRNA RNP complex could perform sequence-specific inhibition of RT reactions.

This study showed that the dCas13a/gRNA RNP complex can be used as a sequence-specific blocker to inhibit RT reactions. This system is potentially useful to examine the targeted binding efficiency of CRISPR/Cas13a in a test tube, independent of cleavage activity. In addition, this system could be used to avoid RT of undesirable or redundant RNA species, such as ribosomal RNA. In this context, CRISPR/Cas13a could be used to cleave target RNA. However, CRISPR/Cas13a cleaves collateral as well as target RNA [5], which may affect RT efficiency. To evaluate the utility of this RT blocking system, other CRISPR/dCas13a orthologs and RT enzymes (kits) could be tested. In addition, endogenous inhibition of RT from the retroviral RNA genome may be an interesting issue from the viewpoint of clinical application.

## Supplementary data

Supplementary data is available at *Biology Methods and Protocols* online.

## Declarations

### Ethics approval and consent to participate

Not applicable.

### Consent for publication

Not applicable.

### Availability of data and materials

All data generated or analyzed from the current study are included in this published article.

## Authors’ contributions

H.F. conceived the project. T.F. designed the experiments. T.F., S.N, and M.Y. performed experiments. T.F. and H.F. wrote the manuscript. All authors read and approved the final manuscript. 

## Funding

This work was supported by the Hirosaki University Graduate School of Medicine and Epigeneron Inc. (T.F. and H.F.).

## Conflict of interest statement

T.F. and H.F. filed a patent for applications of the dCas13a/gRNA RNP complexes including those described in this study. Patent names: “Method for detecting target nucleic acid, Method for detecting molecule having nucleic acid binding ability, and Method for evaluating nucleic acid binding ability.” Patent applicant: Hirosaki University. Name of inventors: T.F. and H.F. Patent numbers: Japanese Patent Application No. 2019-191409; PCT/JP2020/039128. Patent status: filed. Specific aspect of manuscript covered in patent application: the patent application covers application of dCas13a/gRNA RNP complexes to inhibit RT reactions in a sequence-specific manner. T.F. and H.F. are cofounders of Epigeneron Inc. and own stock in the company. T.F. is an advisor and H.F. is a director of Epigeneron, Inc. Epigeneron, Inc. owns the right of commercial use of all filed and approved patents.

## Supplementary Material

bpab009_Supplementary_Data
