# Supplementary material for: Sequence-specific inhibition of reverse transcription by recombinant CRISPR/dCas13a ribonucleoprotein complexes in vitro
Source: Biol Methods Protoc. 2021 Apr 19;6(1):bpab009. doi: 10.1093/biomethods/bpab009 (PMC8106441; doi:10.1093/biomethods/bpab009)
Supplement: bpab009_Supplementary_Data [file bpab009_supplementary_data.zip › 20210407_SupplementaryTableS1.pdf]

Supplementary Table S1

Types	Names	Sequences (5' to 3')	Experiments	Number
gRNA	gRNA_EGFR	gauuuagacuaccccaaaaacgaaggggacuaaaac ccaggccaaaaucugugaucuugacaug	Fig. 3 and Supplementary Fig. S3	-
	gRNA_NEAT1	gauuuagacuaccccaaaaacgaaggggacuaaaac caucaaucugcguuguggcaucaacguu	Fig. 4 and Supplementary Fig. S4	-
	gRNA_NEAT1_2	gauuuagacuaccccaaaaacgaaggggacuaaaac uaucucuaaccaacccucuccccuucuuc	Supplementary Fig. S5	-
Primers	hEGFR-dCas13a-F	CAGGAACGTACTGGTGAAAACAC	Figs. 3 and 4 and Supplementary Figs. S1-S3	28500
	hEGFR-dCas13a-R	CCAGACATCACTCTGGTGGGTAT	Figs. 3 and 4 and Supplementary Figs. S1-S3	28501
	hNEAT1-dCas13a-F	TCCTGGGGTAAAGGTTTTCAGAT	Fig. 4 and Supplementary Fig. S4	28498
	hNEAT1-dCas13a-R	CATCTGCAAGCTCCATCTACAAG	Fig. 4 and Supplementary Fig. S4	28499
	hNEAT1-F4	GTATGCAGCTTGGCACTGGTACT	Supplementary Fig. S5	28732
	hNEAT1-R4	AATTCCCTTCAACCTGCATTT	Supplementary Fig. S5	28733
	hGAPDH-dCas13a-F3	GAGCCAAAAGGGTCATCATCTCT	Figs. 3 and 4 and Supplementary Figs. S3-S5	28502
	hGAPDH-dCas13a-R3	CACGATACCAAAGTTGTCATGGA	Figs. 3 and 4 and Supplementary Figs. S3-S5	28503
