# Supplementary material for: Sequence-specific inhibition of reverse transcription by recombinant CRISPR/dCas13a ribonucleoprotein complexes in vitro
Source: Biol Methods Protoc. 2021 Apr 19;6(1):bpab009. doi: 10.1093/biomethods/bpab009 (PMC8106441; doi:10.1093/biomethods/bpab009)
Supplement: bpab009_Supplementary_Data [file bpab009_supplementary_data.zip › 20210409_SupplementaryText_rev.docx]

**Supplementary Information**

**Sequence-specific inhibition of reverse transcription by recombinant CRISPR/dCas13a ribonucleoprotein complexes *in vitro***

Toshitsugu Fujita, Shoko Nagata, Miyuki Yuno, and Hodaka Fujii

**Supplementary Table and Figure Legends**

**Supplementary Table S1. gRNA and primers used in this study.**

**Supplementary Figure S1. RT in the presence of the dCas13a protein alone.** RT reactions were performed with MRC-5 RNA in the presence of dCas13a protein alone. After RT, cDNA was subjected real-time PCR to amplify the *EGFR* sequence. Amplification plots are shown.

**Supplementary Figure S2. RT in the presence or absence of primers.** (**A**) RT reactions were performed with MRC-5 RNA in the presence or absence of the oligo(dT) primer. The resulting cDNA was subjected to real-time PCR to amplify *EGFR*. Amplification plots are shown. (**B**) Potential models of RT reactions. (a) RT reactions proceed even in the absence of specific primers (primer-independent cDNA synthesis). (b) Primer-dependent cDNA is synthesized by RT in the presence of specific primers such as the oligo(dT) primer. (c) Synthesis of primer-dependent and -independent cDNA is blocked by the sequence-specific dCas13a/gRNA RNP complex. In this model, upstream primers but not a pair of primers between which the target site is located can amplify the target sequence.

**Supplementary Figure S3. Sequence-specific inhibition of RT by the recombinant dCas13a/gRNA_EGFR RNP complex.** (**A**) RT reactions using NCI-H1299 RNA in the presence of the dCas13a/gRNA_EGFR RNP complex. To evaluate RT blocking effects, the cDNA was subjected to real-time PCR to amplify *EGFR* and *GAPDH*. (**B**) Results with normalization to *GAPDH*. Error bars represent the standard deviation of three independent RT experiments (**A** and **B**). *p*-values (*: *p <* 0.05, **: *p <* 0.01, ***: *p <* 0.001) are shown when statistical significance was achieved.

**Supplementary Figure S4. Sequence-specific inhibition of RT by the recombinant dCas13a/gRNA_NEAT1 RNP complex.** (**A**) RT reactions using NCI-H1299 RNA in the presence of the dCas13a/gRNA_NEAT1 RNP complex. To evaluate the RT-blocking effect of the dCas13a/gRNA_NEAT1 RNP complex, the cDNA was subjected to real-time PCR to amplify *NEAT1* and *GAPDH*. (**B**) Results with normalization to *GAPDH*. Error bars represent the standard deviation of three independent RT experiments (**A** and **B**). *p*-values (*: *p <* 0.05, ***: *p <* 0.001) are shown when statistical significance was achieved. In this experiment, 100 ng of total RNA was used because 10 ng was not sufficient to detect *NEAT1*.

**Supplementary Figure S5. Sequence-specific inhibition of RT by the recombinant dCas13a/gRNA_NEAT1_2 RNP complex.** (**A**) Target and primer positions. Primer positions are shown in red, and the cDNA sequence corresponding to the gRNA targeting *NEAT1* RNA is highlighted. (**B**) RT reactions using NCI-H1299 RNA in the presence of the dCas13a/gRNA_NEAT1_2 RNP complex. To evaluate the RT-blocking effect of the dCas13a/gRNA_NEAT1_2 RNP complex, the cDNA was subjected to real-time PCR to amplify *NEAT1* and *GAPDH*. (**C**) Results with normalization to *GAPDH*. Error bars represent the standard deviation of three independent RT experiments (**B** and **C**). *p*-values (*: *p <* 0.05, ***: *p <* 0.001) are shown when statistical significance was achieved. In this experiment, 100 ng of total RNA was used because 10 ng was not sufficient to detect *NEAT1*.
