# Supplementary material for: Sequence-specific inhibition of reverse transcription by recombinant CRISPR/dCas13a ribonucleoprotein complexes in vitro
Source: Biol Methods Protoc. 2021 Apr 19;6(1):bpab009. doi: 10.1093/biomethods/bpab009 (PMC8106441; doi:10.1093/biomethods/bpab009)
Supplement: bpab009_Supplementary_Data [file bpab009_supplementary_data.zip › 20210407_SupplementaryFigure.pdf]

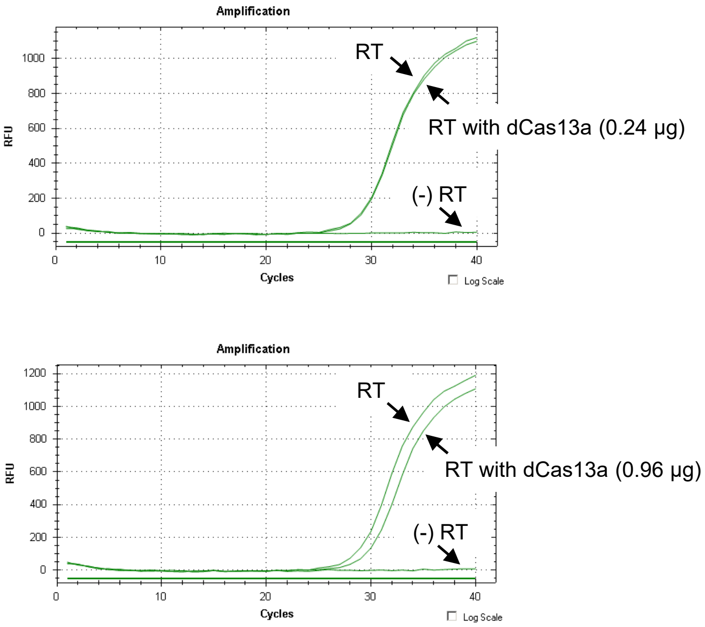

Supplementary Figure S1

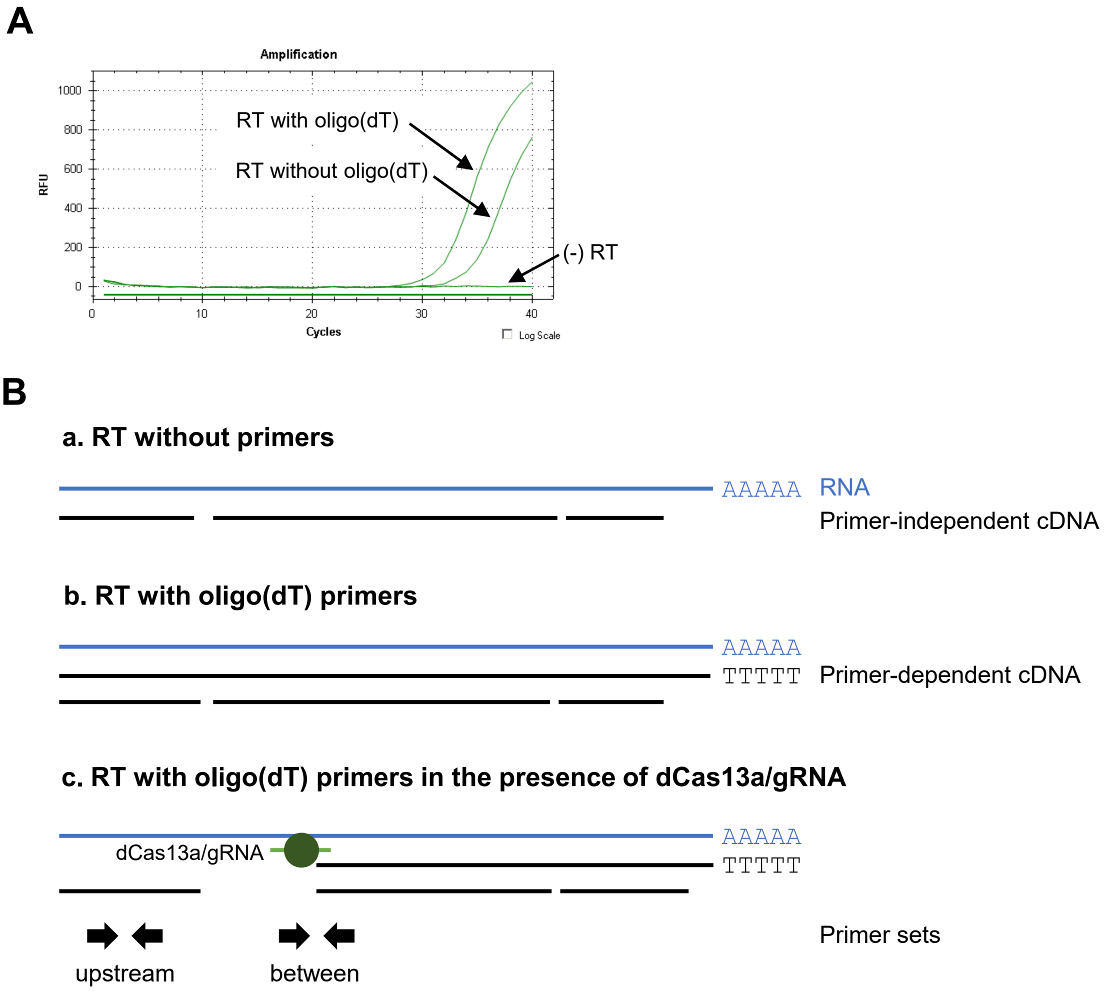

Supplementary Figure S2

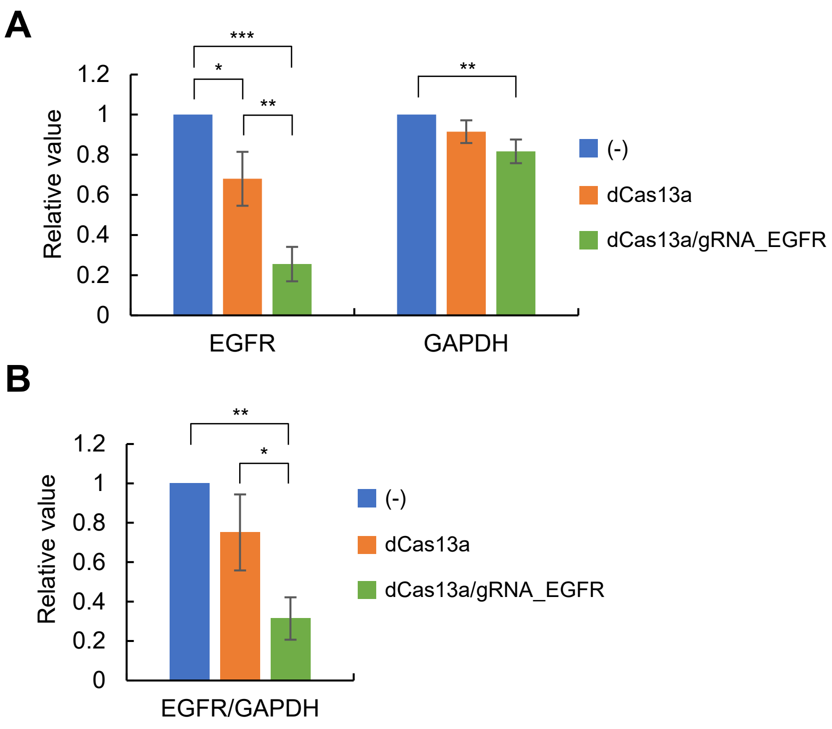

Supplementary Figure S3

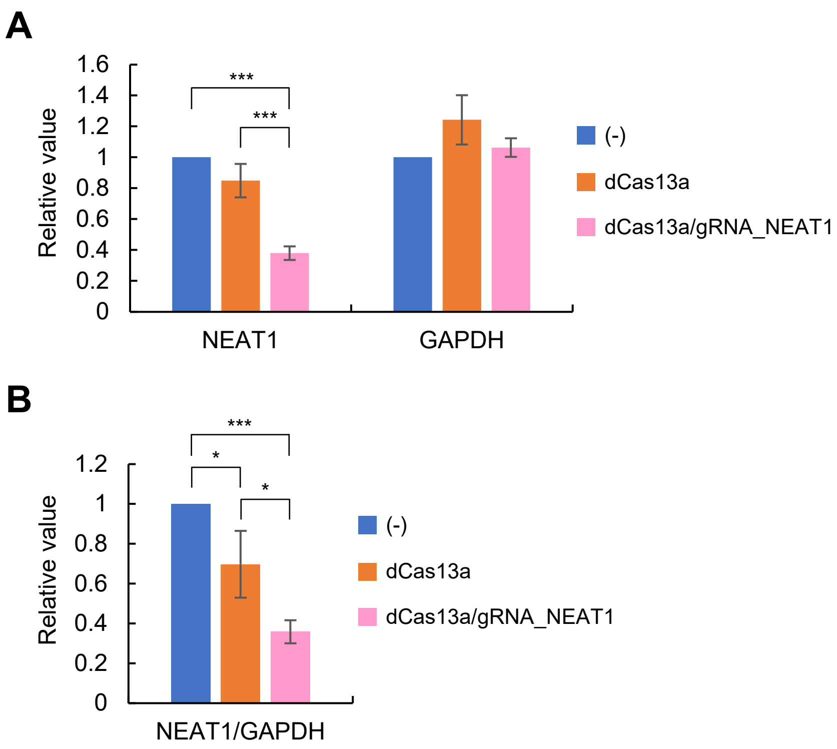

Supplementary Figure S4

A

NEAT1 cDNA (around gRNA_NEAT1_2 target)

5'…gtatgcagcttggcactggtactgggagggatgagggt**gaagaaggggagag**
ggttggttagagatacagtgtgggtggtgggggtggtaggaaatgcaggttgaag
ggaatt…3'

B

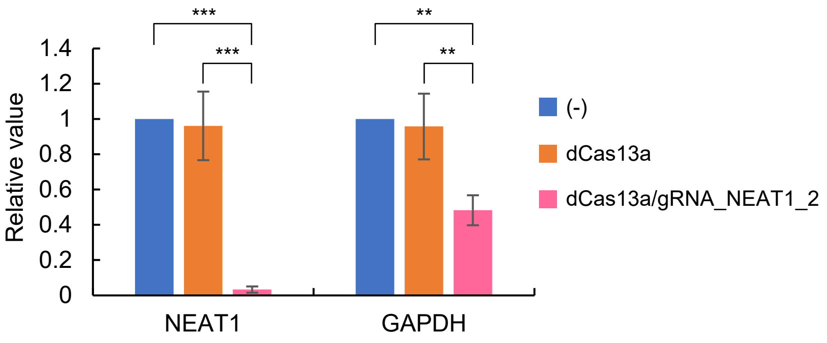

C

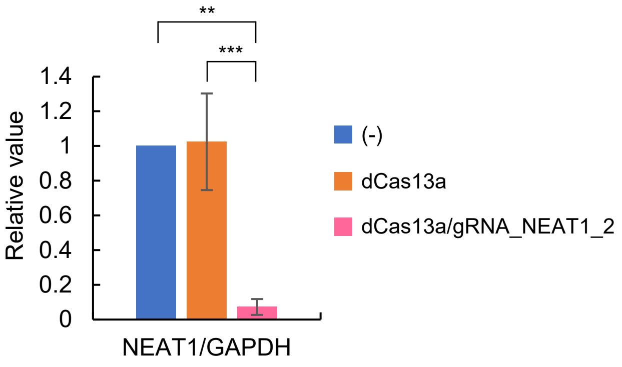

Supplementary Figure S5
